# Is the 90th Percentile Adequate? The Optimal Waist Circumference Cutoff Points for Predicting Cardiovascular Risks in 124,643 15-Year-Old Taiwanese Adolescents

**DOI:** 10.1371/journal.pone.0158818

**Published:** 2016-07-07

**Authors:** Jason Jiunshiou Lee, ChinYu Ho, Hsin-Jen Chen, Nicole Huang, Jade Chienyu Yeh, Sarah deFerranti

**Affiliations:** 1 Department of Family Medicine, Yangming Branch, Taipei City Hospital, Taipei City, Taiwan; 2 Department of Public Health, School of Medicine, National Yang-Ming University, Taipei City, Taiwan; 3 Department of Health Care Management, National Taipei University of Nursing and Health Sciences, Taipei City, Taiwan; 4 Faculty of Medicine, School of Medicine, National Yang-Ming University, Taipei City, Taiwan; 5 Department of Leisure Industry and Health Promotion, National Taipei University of Nursing and Health Sciences, Taipei City, Taiwan; 6 Institute of Hospital and Health Care Administration, National Yang-Ming University, Taipei City, Taiwan; 7 Department of Family Medicine, Lo-Sheng Sanatorium and Hospital, Ministry of Health and Welfare, New Taipei City, Taiwan; 8 Department of Cardiology, Boston Children's Hospital, Boston, United States of America; Institute of Preventive Medicine, DENMARK

## Abstract

Adolescent obesity has increased to alarming proportions globally. However, few studies have investigated the optimal waist circumference (WC) of Asian adolescents. This study sought to establish the optimal WC cutoff points that identify a cluster of cardiovascular risk factors (CVRFs) among 15-year-old ethnically Chinese adolescents. This study was a regional population-based study on the CVRFs among adolescents who enrolled in all the senior high schools in Taipei City, Taiwan, between 2011 and 2014. Four cross-sectional health examinations of first-year senior high school (grade 10) students were conducted from September to December of each year. A total of 124,643 adolescents aged 15 (boys: 63,654; girls: 60,989) were recruited. Participants who had at least three of five CVRFs were classified as the high-risk group. We used receiver-operating characteristic curves and the area under the curve (AUC) to determine the optimal WC cutoff points and the accuracy of WC in predicting high cardiovascular risk. WC was a good predictor for high cardiovascular risk for both boys (AUC: 0.845, 95% confidence interval [CI]: 0.833–0.857) and girls (AUC: 0.763, 95% CI: 0.731–0.795). The optimal WC cutoff points were ≥78.9 cm for boys (77th percentile) and ≥70.7 cm for girls (77th percentile). Adolescents with normal weight and an abnormal WC were more likely to be in the high cardiovascular risk group (odds ratio: 3.70, 95% CI: 2.65–5.17) compared to their peers with normal weight and normal WC. The optimal WC cutoff point of 15-year-old Taiwanese adolescents for identifying CVRFs should be the 77^th^ percentile; the 90^th^ percentile of the WC might be inadequate. The high WC criteria can help health professionals identify higher proportion of the adolescents with cardiovascular risks and refer them for further evaluations and interventions. Adolescents’ height, weight and WC should be measured as a standard practice in routine health checkups.

## Introduction

Childhood and adolescent obesity has increased to alarming proportions globally because of sedentary lifestyles and unhealthy dietary habits in both developed and developing countries [1–4]. Obesity has short- and long-term physical and psychological consequences [4–6], and overweight children have significantly higher odds of remaining obese into adulthood and a greater risk of developing cardiovascular diseases later in life [6–9].

Abdominal obesity is associated with a higher risk of diabetes and cardiovascular disease [10–12]. The waist circumference (WC) can reflect the true amount of intraabdominal fat, whereas a higher body mass index (BMI, weight in kilograms divided by height in meters squared, kg m^-2^) may be due to increased muscle mass [13]. The WC standards for adults differ among countries. The U.S. National Institutes of Health guidelines suggest that the WC thresholds of cardio-metabolic risk in adults are 102 cm for men and 88 cm for women [14–17]. In Asian countries, the thresholds are 90 cm and 80 cm for men and women, respectively [18]. The WC standards are also different for adolescents in different ethnicities and age groups [19]. The International Diabetes Federation (IDF) consensus definition of metabolic syndrome in children and adolescents designates the 90th percentile of WC as the cutoff point for defining central obesity, with ethnicity-specific values for European-American, African-American, and Mexican-American populations [20–22].

Some studies have investigated the association between the WC and cardiovascular disease in various ethnicities, but little information on Asian adolescents is available. Moreover, the IDF consensus does not recommend a standard for Asian-Americans. The purpose of this study was to establish the optimal WC cutoff points that identify a cluster of cardiovascular risk factors among a 15-year-old ethnically Chinese adolescent population and to investigate the odds of having the cardiovascular risk factors for different BMI and WC categories. The high WC criteria can help health professionals identify higher proportion of the adolescents with cardiovascular risks and refer them for further evaluations and interventions.

## Methods

### Study population

It has been a health policy that Taipei City government holds health examinations for all first-year senior high school (grade 10) students in Taipei City every year since 2011. Every student’s parents have to agree their children for the health checkups and blood tests. Our study was a regional population-based study on the cardiovascular risk factors among adolescents who enrolled in all the senior high schools in Taipei City, Taiwan, between 2011 and 2014. Four cross-sectional health examinations of these students were conducted by Taipei City Hospital from September to December of each year. There were 46,182, 43,115, 40,241, and 36,595 first-year students enrolled in all the senior high schools in Taipei City in 2011, 2012, 2013, and 2014, respectively.

Our cross-sectional analyses retrospectively collected anonymous data from Taipei City Hospital and focused on 15-year-old adolescents who were fasting for at least 8 hours. Each subject’s height, weight, waist circumference, systolic and diastolic blood pressure, fasting glucose, total cholesterol, triglyceride, and high-density lipoprotein (HDL) cholesterol levels were recorded. Of the 166,133 first-year students of all the senior high schools in Taipei City between 2011 and 2014, our study was limited to 131,565 adolescents who were 15 years old; 128,446 participants (97.63%) were ultimately enrolled in the study. The final analysis included 124,643 adolescents aged 15 (boys: 63,654; girls: 60,989) because of the following exclusive reasons: without fasting status (n = 3,723) and implausible data (n = 80). The institutional Review Board of Taipei City Hospital (TCHIRB-1000908, TCHIRB-1010902) approved this study to retrospectively collect the anonymous data and the investigation.

### Measurements

The anthropometric parameters used included body height, weight, and WC. Each subject was shoeless and wore a thin layer of clothes, and well-trained technicians measured their height and weight by using a NAGATA P120WH measuring machine. The WC was measured at the midpoint between the lowest rib and the iliac crest by using non-elastic measuring tape identical in all measurements (WHO protocol, Report of the WHO Expert Consultation: Waist Circumference and Waist–Hip Ratio, Geneva, 8–11 December 2008) [23]. Before the WC measurement, the technician asked the student to take a few deep and natural breaths. During the actual measurement, the student who has fasted overnight was asked to stand a relaxed posture with arms at the sides, feet positioned close together and weight evenly distributed across the feet. The technician measured the student’s WC at the end of his/her normal expiration. Each seated subject’s systolic blood pressure (SBP) and diastolic blood pressure (DBP) were measured in the right arm after at least 10 minutes of rest by using an automatic sphygmomanometer (TERUMO ESP2000). After fasting overnight for 8 hours, each student had blood drawn by clinical laboratory technologist using a standard venipuncture technique. Fasting glucose, total cholesterol, triglyceride and HDL cholesterol levels were measured at the central laboratory of Taipei City Hospital.

The cardiovascular risk factors included in the analysis were elevated SBP or DBP (SBP ≥120 mmHg or DBP ≥80 mmHg) [24], impaired fasting glucose (≥100 mg dL^-1^) and an abnormal lipid profile [25]. According to the IDF definition, lipid abnormalities of an individual aged 10–15 years were defined as total cholesterol ≥200 mg dL^-1^, triglyceride ≥150 mg dL^-1^, and HDL cholesterol <40 mg dL^-1^. Participants who had at least three risk factors were classified into the high-risk group, whereas those who had fewer than three risk factors were categorized into the low-risk group. Overweight and obese adolescents were identified using the sex- and age-specific BMI criteria from the Department of Health (DOH) in Taiwan [26], where the BMI of a 15-year-old boy from 22.9 to 25.3 indicates overweight and a BMI ≥25.4 indicates obese. The BMI of a 15-year-old girl from 22.7 to 25.1 indicates overweight and a BMI ≥25.2 indicates obese.

### Statistical Analysis

Statistical procedures were performed using Statistical Analysis Systems version 9.4 (SAS Institute, Cary, NC). We used receiver-operating characteristic (ROC) curves and the area under the ROC curve (AUC) to measure the accuracy of the WC. We determined the optimal WC cutoff point by choosing the point that had the shortest distance from the ROC curve to the upper-left corner point (the ideal point). A perfect test shows 1.0 of its AUC value, an area of 0.5–0.74 is considered a fair test, and an area of 0.75–0.92 is effectively a good test [27].

We categorized all subjects into six BMI-WC groups using the optimal WC cutoff points that we determined, in addition to the BMI standard from the DOH in Taiwan [26]. The participants were separated into the following six groups: normal weight with a normal WC, normal weight with an abnormal WC, overweight with a normal WC, overweight with an abnormal WC, obese with a normal WC, and obese with an abnormal WC. Logistic regression was performed with all five cardiovascular risk factors as the dependent variables to determine the association between the risk factors and these six groups after controlling for sex.

## Results

The subject characteristics are shown in Table 1. The average ages of boys and girls among the 124,643 participants were 15.58 and 15.57, respectively. Among the 63,654 15-year-old boys, 956 (1.50%) of them were categorized into the high cardiovascular risk group, whereas 308 of the 60,989 15-year-old girls (0.51%) were categorized into the high cardiovascular risk group (Table 2).

**Table 1:** Characteristics of participants.

	15 year-old (N = 124,643)		
	Male (N = 63,654)	Female (N = 60,989)	
	Mean ± S.D.	Mean ± S.D.	p value
Age (years)	15.58 ± 0.26	15.57 ± 0.26	<0.0001
Height (cm)	170.4 ± 6.1	159.1 ± 5.5	<0.0001
Weight (kg)	63.5 ± 13.7	52.6 ± 9.6	<0.0001
Body Mass Index (kg m^-2^)	21.8 ± 4.3	20.7 ± 3.5	<0.0001
Waist Circumference (cm)	72.6 ± 10.8	66.6 ± 8.1	<0.0001
Systolic Blood Pressure (mmHg)	118.6 ± 14.4	108.2 ± 13.3	<0.0001
Diastolic Blood Pressure (mmHg)	62.8 ± 10.6	64.3 ± 9.7	<0.0001
Fasting Glucose (mg dL^-1^)	85.4 ± 9.6	83.7 ± 9.3	<0.0001
Total Cholesterol (mg dL^-1^)	153.9 ± 26.8	166.6 ± 28.2	<0.0001
Triglyceride (mg dL^-1^)	71.7 ± 35.4	69.2 ± 28.0	<0.0001
HDL cholesterol (mg dL^-1^)	56.9 ± 11.6	64.1 ± 12.8	<0.0001

The data on every characteristic were available for all participants and these continuous data were analyzed using unpaired t test.

Abbreviation: HDL, high-density lipoprotein.

**Table 2:** Numbers of participants having cardiovascular risk factors.

	15 y (N = 124,643)	
	Male (N = 63,654)	Female (60,989)
Numbers of cardiovascular risk factors	N (%)	N (%)
<2	57,927 (91.00)	58,104 (95.27)
2	4,771 (7.50)	2,577 (4.23)
3	829 (1.30)	276 (0.45)
4	122 (0.19)	31 (0.05)
5	5 (0.01)	1 (<0.01)

**Fig 1:**
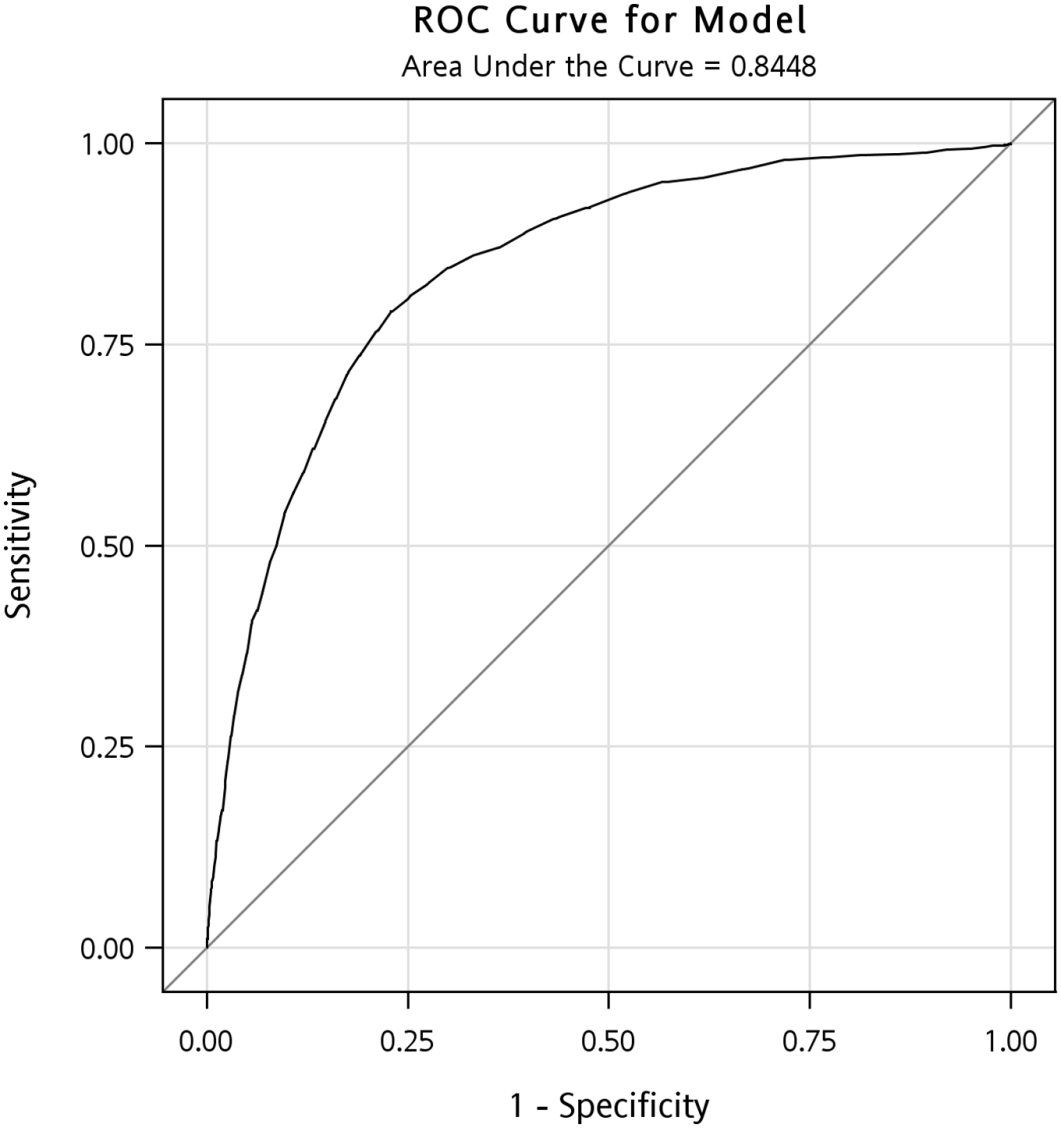
The ROC curve for the 15-year-old boys. The AUC identified in ROC analysis for the boys was 0.845 (95% CI: 0.833–0.857).

**Fig 2:**
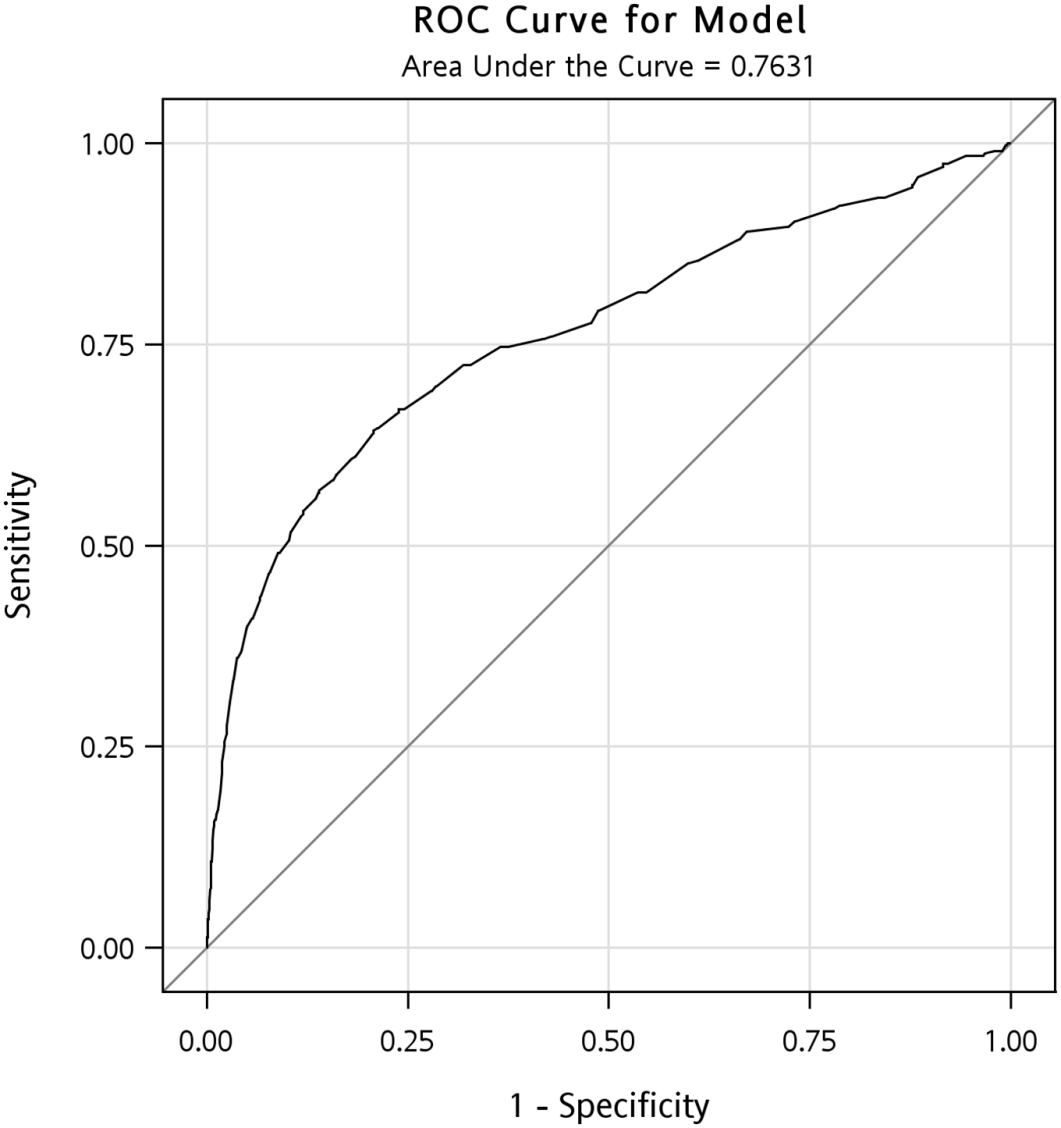
The ROC curve for the 15-year-old girls. The AUC identified in ROC analysis for the girls was 0.763 (95% CI: 0.731–0.795).

The AUCs identified in ROC analysis for the boys and girls were 0.845 (95% confidence interval [CI]: 0.833–0.857) and 0.763 (95% CI: 0.731–0.795) (Figs 1 and 2). Determining the shortest distance from the ROC curve to the upper-left corner ideal point indicated that the optimal WC cutoff points are ≥78.9 cm for 15-year-old boys (the 77th percentile; sensitivity 79.1%, specificity 77.1%, positive predictive value 5.0%, negative predictive value 99.6%) and ≥70.7 cm for 15-year-old girls (the 77th percentile; sensitivity 66.9%, specificity 76.1%, positive predictive value 1.4%, negative predictive value 99.8%). By using the cutoff point of the 90th percentile, the positive predictive value for boys and girls was 7.84% and 2.46%, respectively. However, in comparison with using the cutoff point of the 77th and 90th percentile, the sensitivity declined from 79.1% to 53.8% for boys and 66.9% to 50.7% for girls. Health professionals are able to detect more proportion of the students with cardiovascular risks while using the 77th cutoff points.

Adolescents with a WC above the optimal cutoff point were more likely to have cardiovascular risk factors than those with a normal WC after controlling for BMI and sex (Table 3). Compared with those who were of normal weight and had a normal WC after we controlled for sex, the adolescents with normal weight and an abnormal WC were more likely to be in the high cardiovascular risk group (odds ratio [OR]: 3.70, 95% CI: 2.65–5.17). Overweight adolescents with an abnormal WC and obese adolescents with an abnormal WC were more likely to be in the high cardiovascular risk group (OR: 5.40, 95% CI: 4.26–6.84) (OR: 18.23, 95% CI: 15.59–21.32) than those who were of normal weight and had a normal WC, respectively (Table 4). Notably, the adolescents with an abnormal WC had higher odds of being in the high cardiovascular risk group, having higher cholesterol, glucose and triglyceride levels than those of adolescents with a normal WC, even when the BMI was higher.

**Table 3:** The odds ratio (OR) of the different BMI and WC categories having the cardiovascular risk factors.

	High CV Risk Group^a^	Elevated BP^b^	Abnormal cholesterol^c^	Abnormal glucose^d^	Abnormal triglyceride^e^	Abnormal HDL^f^
Characteristic	OR (95%CI)	OR (95%CI)	OR (95%CI)	OR (95%CI)	OR (95%CI)	OR (95%CI)
Normal weight^g^	1.00 (reference)	1.00 (reference)	1.00 (reference)	1.00 (reference)	1.00 (reference)	1.00 (reference)
Overweight^h^	2.04 (1.62–2.56)	1.40 (1.34–1.45)	1.13 (1.05–1.21)	1.00 (0.90–1.12)	1.74 (1.53–1.98)	1.97 (1.76–2.21)
Obese^i^	5.83 (4.66–7.31)	2.11 (2.00–2.21)	1.72 (1.59–1.86)	1.49 (1.31–1.69)	4.09 (3.59–4.67)	4.12 (3.64–4.67)
Normal WC	1.00 (reference)	1.00 (reference)	1.00 (reference)	1.00 (reference)	1.00 (reference)	1.00 (reference)
Abnormal WC^j^	2.98 (2.42–3.66)	1.32 (1.27–1.38)	1.25 (1.17–1.34)	1.30 (1.16–1.45)	2.51 (2.23–2.83)	1.51 (1.35–1.69)
Female	1.00 (reference)	1.00 (reference)	1.00 (reference)	1.00 (reference)	1.00 (reference)	0.36 (0.33–0.39)
Male	2.32 (2.03–2.66)	3.43 (3.34–3.52)	0.43 (0.41–0.45)	1.66 (1.55–1.78)	1.78 (1.64–1.93)	1.00 (reference)

Abbreviation: OR: odds ratio; BMI: body mass index; WC: waist circumference; CV: cardiovascular; BP: blood pressure; SBP: systolic blood pressure; DBP: diastolic blood pressure; HDL: high-density lipoprotein; IDF: International Diabetes Federation.

The five cardiovascular risk factors were elevated BP, abnormal cholesterol, abnormal glucose, abnormal triglyceride and abnormal HDL, and participants who had at least three risk factors were classified into the

^a^high CV risk group.

^b^Elevated BP was defined as SBP ≥120 mmHg or DBP ≥80 mmHg.

^c^Abnormal cholesterol was defined by the IDF as total cholesterol ≥200 mg dL^-1^.

^d^Abnormal glucose was defined by the IDF as fasting glucose ≥100 mg dL^-1^.

^e^Abnormal triglyceride was defined by the IDF as triglyceride ≥150 mg dL^-1^.

^f^Abnormal HDL was defined by the IDF as HDL cholesterol <40 mg dL^-1^.

Normal weight, overweight and obese adolescents were identified using the sex- and age-specific BMI criteria from the Department of Health in Taiwan, where the BMI of a 15-year-old boy from 16.9 to 22.8 indicates ^g^normal weight, a BMI from 22.9 to 25.3 indicates ^h^overweight and a BMI ≥25.4 indicates ^i^obese. The BMI of a 15-year-old girl from 16.7 to 22.6 indicates

^g^normal weight, a BMI from 22.7 to 25.1 indicates

^h^overweight and a BMI ≥25.2 indicates

^i^obese.

^j^Abnormal WC was defined as the WC ≥ the optimal cutoff point (the 77^th^ percentile) this study determined.

The independent variables used in regression were BMI, WC and sex. Logistic regression was performed with all five cardiovascular risk factors as the dependent variables to determine the association between the risk factors, BMI and WC after controlling for sex among 15-year-old Taiwanese adolescents.

**Table 4:** The odds ratio (OR) of the six BMI-WC groups having the cardiovascular risk factors.

	High CV Risk Group^a^	Elevated BP^b^	Abnormal cholesterol^c^	Abnormal glucose^d^	Abnormal triglyceride^e^	Abnormal HDL^f^
Characteristic	OR (95%CI)	OR (95%CI)	OR (95%CI)	OR (95%CI)	OR (95%CI)	OR (95%CI)
Normal weight^g^ with normal WC	1.00 (reference)	1.00 (reference)	1.00 (reference)	1.00 (reference)	1.00 (reference)	1.00 (reference)
Normal weight^g^ with abnormal WC^j^	3.70 (2.65–5.17)	1.29 (1.21–1.37)	1.30 (1.19–1.41)	1.21 (1.02–1.43)	2.55 (2.10–3.08)	1.99 (1.64–2.41)
Overweight^h^ with normal WC	2.83 (2.15–3.74)	1.43 (1.37–1.50)	1.25 (1.14–1.36)	1.01 (0.88–1.16)	1.91 (1.62–2.26)	2.26 (1.98–2.57)
Overweight^h^ with abnormal WC^j^	5.40 (4.26–6.84)	1.79 (1.71–1.89)	1.32 (1.22–1.43)	1.29 (1.13–1.47)	4.16 (3.64–4.75)	2.73 (2.39–3.12)
Obese^i^ with normal WC	3.76 (2.29–6.20)	1.67 (1.50–1.86)	1.25 (1.02–1.54)	1.18 (0.88–1.58)	3.22 (2.43–4.28)	3.99 (3.22–4.95)
Obese^i^ with abnormal WC^j^	18.23 (15.59–21.32)	2.84 (2.74–2.95)	2.20 (2.09–2.33)	1.96 (1.80–2.12)	10.39 (9.53–11.34)	6.38 (5.89–6.91)
Female	1.00 (reference)	1.00 (reference)	1.00 (reference)	1.00 (reference)	1.00 (reference)	0.35 (0.33–0.38)
Male	2.32 (2.03–2.66)	3.42 (3.34–3.52)	0.43 (0.41–0.44)	1.65 (1.54–1.78)	1.78 (1.64–1.92)	1.00 (reference)

Abbreviation: OR: odds ratio; BMI: body mass index; WC: waist circumference; CV: cardiovascular; BP: blood pressure; SBP: systolic blood pressure; DBP: diastolic blood pressure; HDL: high-density lipoprotein; IDF: International Diabetes Federation.

The five cardiovascular risk factors were elevated BP, abnormal cholesterol, abnormal glucose, abnormal triglyceride and abnormal HDL, and participants who had at least three risk factors were classified into the

^a^high CV risk group.

^b^Elevated BP was defined as SBP ≥120 mmHg or DBP ≥80 mmHg.

^c^Abnormal cholesterol was defined by the IDF as total cholesterol ≥200 mg dL^-1^.

^d^Abnormal glucose was defined by the IDF as fasting glucose ≥100 mg dL^-1^.

^e^Abnormal triglyceride was defined by the IDF as triglyceride ≥150 mg dL^-1^.

^f^Abnormal HDL was defined by the IDF as HDL cholesterol <40 mg dL^-1^.

Normal weight, overweight and obese adolescents were identified using the sex- and age-specific BMI criteria from the Department of Health in Taiwan, where the BMI of a 15-year-old boy from 16.9 to 22.8 indicates

^g^normal weight, a BMI from 22.9 to 25.3 indicates

^h^overweight and a BMI ≥25.4 indicates ^i^obese. The BMI of a 15-year-old girl from 16.7 to 22.6 indicates ^g^normal weight, a BMI from 22.7 to 25.1 indicates ^h^overweight and a BMI ≥25.2 indicates

^i^obese.

^j^Abnormal WC was defined as the WC ≥ the optimal cutoff point (the 77^th^ percentile) this study determined.

The independent variables used in regression were BMI-WC groups and sex. Logistic regression was performed with all five cardiovascular risk factors as the dependent variables to determine the association between the risk factors and these six groups after controlling for sex among 15-year-old Taiwanese adolescents.

### Sensitivity test

Previous studies have used different criteria for defining the high cardiovascular risk group; for example, some studies have defined the high-risk group as adolescents who had at least two risk factors [27], whereas other studies have used three risk factors as the criterion. If we alter the definition of the high-risk group from having at least three risk factors to having at least two risk factors in this study, there would be 5,727 (9.00%) boys and 2,885 (4.73%) girls categorized into the high-risk group. The AUCs identified in ROC analysis for the boys and girls were 0.730 (95% CI: 0.723–0.737) and 0.638 (95% CI: 0.626–0.649). The optimal WC cutoff points were ≥76.5 cm for 15-year-old boys (the 72nd percentile; sensitivity 61.1%, specificity 74.7%, positive predictive value 19.3%, negative predictive value 95.1%) and ≥70.7 cm for 15-year-old girls (the 77th percentile; sensitivity 45.2%, specificity 77.0%, positive predictive value 8.9%, negative predictive value 96.6%).

By using the IDF criteria of abnormal blood pressure (SBP≥130 mmHg or DBP≥85 mmHg) [25], the AUCs identified in ROC analysis for the boys and girls were 0.866 (95% confidence interval [CI]: 0.853–0.879) and 0.784 (95% CI: 0.741–0.826). The optimal WC cutoff points are ≥78.9 cm for 15-year-old boys (the 77th percentile; sensitivity 83.0%, specificity 76.9%) and ≥71.9 cm for 15-year-old girls (the 79th percentile; sensitivity 69.6%, specificity 79.2%).

## Discussion

Ours was the first large-sample population-based study to investigate the optimal WC cutoff point for identifying high cardiovascular risk among Asian adolescents. The AUCs showed that the WC is a clear indicator for identifying and predicting the cardiovascular risk factors among the 15-year-old ethnically Chinese adolescent population. The prevalence proportions of having three cardiovascular risk factors among the adolescents in our study were 1.50% and 0.51% for the boys and girls, respectively. Similar to a study that analyzed an NHANES (National Health and Nutrition Examination Survey) III data set collected between 1988 and 1994 on non-Hispanic white, non-Hispanic black, and Hispanic ethnicities in the United States [27], we determined that the WC is more accurate for predicting cardiovascular risk factors in male than in female adolescents.

We determined that adolescents who are in the same BMI category but have a WC above the optimal cutoff point are more likely to have multiple cardiovascular risk factors. The adolescents’ WC is a clearer indicator than the BMI, and could provide more information about the cardiovascular risk factors in our study population. Our study mainly focused on ethnically Chinese adolescents, yet the conclusion is comparable to that of other studies that have focused on other ethnic populations. Previous studies have demonstrated that the WC is a more accurate predictor of abnormal blood pressure, HDL cholesterol, and low-density lipoprotein than the BMI in European boys and girls [28, 29]. In the Bogalusa Heart Study, the WC or waist-to-hip ratio level was predictive of abnormal lipid profiles and insulin levels, and correlated with skinfold thickness as a measure of adiposity [29, 30]. For the NHANES III survey, the investigators found that a WC above the identified cutoff point is associated with an increased risk of cardio-metabolic disease in normal-weight male adolescents and an increased risk of elevated blood pressure in overweight female adolescents [27]. Although WC percentile does not show strong evidence as superior screening tools compared with BMI percentile among 10–13 years old adolescents [31], we found that 15-year-old ethnically Chinese adolescents with an abnormal WC had higher odds of elevated blood pressure, higher fasting glucose, cholesterol, triglyceride levels, and lower HDL levels than those of adolescents with a normal WC in all BMI categories.

People with metabolic syndrome—a collection of medical disorders—have a higher risk of developing cardiovascular disease and diabetes mellitus compared with those without the syndrome [32]. Because of the growing epidemic in the young population, the prevalence of childhood metabolic syndrome is also increasing rapidly [33]. The standard cutoff for abnormal WC in adolescent metabolic syndrome defined by the IDF is the 90th percentile; however, the optimal WC cutoff point for identifying the cluster of cardiovascular risk factors in our study was considerably lower. Our results are close to those of other studies indicating the 75th percentile as the threshold for abdominal obesity and cutoff point for cardio-metabolic risk among adolescents [34, 35]. Although the IDF has designated the 90th percentile as the WC cutoff point, they still mentioned that the cutoff point should be reassessed as more evidence becomes available. Therefore, adolescents whose WCs are between the 75th and 90th percentiles should be aware of the risk of cardiovascular diseases.

One of the strengths of this study is that this was the first study to focus on first-year senior high school adolescents in an Asian country. In addition, we established the optimal WC cutoff points of a specific age and sex that can identify a cluster of cardiovascular risk factors for ethnically Chinese adolescents. The sample size used in this study was large with a high participation rate (97.63%). Two physicians separately reviewed the data and excluded implausible records. Furthermore, the WC cutoff points found in this study were lower than those of non-Asian adolescents; the same applies to the adult WC cutoff points in Asia compared with those of Western countries. We also determined that increasing odds of having cardiovascular risk factors among six categories indicate the importance of measuring the WC instead of the BMI. The primary limitation of this cross-sectional study is that because the WC measurement and the cardiovascular risk factors are simultaneously assessed, there is no evidence of a temporal relationship. However, obesity is well known an independent risk factor for cardiovascular diseases, and cardiovascular risks have also been documented in obese adolescents [36]. Moreover, this study was conducted in Taipei City, and the study participants might have been less physically active than those living in rural areas of Taiwan. A nationwide survey of cardiovascular risk factors among all adolescent age groups would reduce this limitation and provide more information on the WC cutoff points of other age groups.

In conclusion, the AUCs showed that the WC is a clear indicator for identifying and predicting the cardiovascular risk factors among the 15-year-old Taiwanese adolescent population. Furthermore, the optimal cutoff point of 15-year-old adolescents for early detection of the risk of cardiovascular disease risk factors is the 77th percentile of the WC. The 90th percentile of the WC might be inadequate. Moreover, we should use both of WC and BMI measurements for predicting cardiovascular risk factors among adolescents. Adolescents who have an abnormal WC in any BMI categories have higher odds of having cardiovascular risk factors. The high WC criteria can help health professionals identify higher proportion of the adolescents with cardiovascular risks and refer them for further evaluations and interventions. Adolescents’ WC and BMI should be measured together as a standard practice in routine health checkups [37]. Future research may consider conducting a longitudinal follow-up study on the same cohort in a nationwide database.

## References

[1] Lakshman R, Elks CE, Ong KK. Childhood obesity. Circulation. 2012;126(14):1770–9. 10.1161/CIRCULATIONAHA.111.047738 23027812 PMC3785130

[2] Wofford LG. Systematic review of childhood obesity prevention. J Pediatr Nurs. 2008;23(1):5–19. 10.1016/j.pedn.2007.07.006 . 18207043

[3] Ogden CL, Carroll MD, Kit BK, Flegal KM. Prevalence of obesity and trends in body mass index among US children and adolescents, 1999–2010. JAMA. 2012;307(5):483–90. 10.1001/jama.2012.40 . 22253364 PMC6362452

[4] Popkin BM, Adair LS, Ng SW. Global nutrition transition and the pandemic of obesity in developing countries. Nutr Rev. 2012;70(1):3–21. 10.1111/j.1753-4887.2011.00456.x 22221213 PMC3257829

[5] Ebbeling CB, Pawlak DB, Ludwig DS. Childhood obesity: public-health crisis, common sense cure. Lancet. 2002;360(9331):473–82. 10.1016/S0140-6736(02)09678-2 . 12241736

[6] Whitaker RC, Wright JA, Pepe MS, Seidel KD, Dietz WH. Predicting obesity in young adulthood from childhood and parental obesity. N Engl J Med. 1997;337(13):869–73. 10.1056/NEJM199709253371301 . 9302300

[7] Singh AS, Mulder C, Twisk JW, van Mechelen W, Chinapaw MJ. Tracking of childhood overweight into adulthood: a systematic review of the literature. Obes Rev. 2008;9(5):474–88. Epub 2008/03/12. 10.1111/j.1467-789X.2008.00475.x . 18331423

[8] Baker JL, Olsen LW, Sorensen TI. Childhood body-mass index and the risk of coronary heart disease in adulthood. N Engl J Med. 2007;357(23):2329–37. 10.1056/NEJMoa072515 18057335 PMC3062903

[9] Leibowitz KL, Moore RH, Stunkard AJ, Stallings VA, Berkowitz RI, Stettler N, et al Cardiovascular disease risk factor (CVDRF) associated waist circumference patterns in obese-prone children. Int J Pediatr Obes. 2009;4(3):150–9. 10.1080/17477160802596130 . 19101853

[10] Despres JP. Intra-abdominal obesity: an untreated risk factor for Type 2 diabetes and cardiovascular disease. J Endocrinol Invest. 2006;29(3 Suppl):77–82. . 16751711

[11] Maffeis C, Pietrobelli A, Grezzani A, Provera S, Tato L. Waist circumference and cardiovascular risk factors in prepubertal children. Obes Res. 2001;9(3):179–87. 10.1038/oby.2001.19 . 11323443

[12] O'Neill S, O'Driscoll L. Metabolic syndrome: a closer look at the growing epidemic and its associated pathologies. Obes Rev. 2015;16(1):1–12. 10.1111/obr.12229 . 25407540

[13] Clasey JL, Bouchard C, Teates CD, Riblett JE, Thorner MO, Hartman ML, et al The use of anthropometric and dual-energy X-ray absorptiometry (DXA) measures to estimate total abdominal and abdominal visceral fat in men and women. Obes Res. 1999;7(3):256–64. . 10348496 10.1002/j.1550-8528.1999.tb00404.x

[14] Lean ME, Han TS, Morrison CE. Waist circumference as a measure for indicating need for weight management. BMJ. 1995;311(6998):158–61. Epub 1995/07/15. 7613427 10.1136/bmj.311.6998.158 PMC2550221

[15] Janssen I, Katzmarzyk PT, Ross R. Body mass index, waist circumference, and health risk: evidence in support of current National Institutes of Health guidelines. Arch Intern Med. 2002;162(18):2074–9. Epub 2002/10/11. . 12374515 10.1001/archinte.162.18.2074

[16] Clinical Guidelines on the Identification, Evaluation, and Treatment of Overweight and Obesity in Adults—The Evidence Report. National Institutes of Health. Obes Res. 1998;6 Suppl 2:51S–209S. Epub 1998/11/14. . 9813653

[17] Zhu S, Wang Z, Heshka S, Heo M, Faith MS, Heymsfield SB. Waist circumference and obesity-associated risk factors among whites in the third National Health and Nutrition Examination Survey: clinical action thresholds. Am J Clin Nutr. 2002;76(4):743–9. . 12324286 10.1093/ajcn/76.4.743

[18] Consultation WE. Appropriate body-mass index for Asian populations and its implications for policy and intervention strategies. Lancet. 2004;363(9403):157–63. Epub 2004/01/17. 10.1016/s0140-6736(03)15268-3 . 14726171

[19] Katzmarzyk PT, Bray GA, Greenway FL, Johnson WD, Newton RL Jr., Ravussin E, et al Ethnic-specific BMI and waist circumference thresholds. Obesity (Silver Spring). 2011;19(6):1272–8. 10.1038/oby.2010.319 21212770 PMC3933952

[20] Anderson PJ, Critchley JA, Chan JC, Cockram CS, Lee ZS, Thomas GN, et al Factor analysis of the metabolic syndrome: obesity vs insulin resistance as the central abnormality. Int J Obes Relat Metab Disord. 2001;25(12):1782–8. Epub 2002/01/10. 10.1038/sj.ijo.0801837 . 11781758

[21] Hirschler V, Aranda C, Calcagno Mde L, Maccalini G, Jadzinsky M. Can waist circumference identify children with the metabolic syndrome? Arch Pediatr Adolesc Med. 2005;159(8):740–4. Epub 2005/08/03. 10.1001/archpedi.159.8.740 . 16061781

[22] Fernandez JR, Redden DT, Pietrobelli A, Allison DB. Waist circumference percentiles in nationally representative samples of African-American, European-American, and Mexican-American children and adolescents. J Pediatr. 2004;145(4):439–44. Epub 2004/10/14. 10.1016/j.jpeds.2004.06.044 . 15480363

[23] Waist circumference and waist–hip ratio: report of a WHO expert consultation, Geneva, 8–11 12 2008: World Health Organization.

[24] Falkner B, Daniels SR. Summary of the Fourth Report on the Diagnosis, Evaluation, and Treatment of High Blood Pressure in Children and Adolescents. Hypertension. 2004;44(4):387–8. 10.1161/01.HYP.0000143545.54637.af . 15353515

[25] Zimmet P, Alberti KG, Kaufman F, Tajima N, Silink M, Arslanian S, et al The metabolic syndrome in children and adolescents—an IDF consensus report. Pediatr Diabetes. 2007;8(5):299–306. 10.1111/j.1399-5448.2007.00271.x . 17850473

[26] Chen W, Chang MH. New growth charts for Taiwanese children and adolescents based on World Health Organization standards and health-related physical fitness. Pediatr Neonatol. 2010;51(2):69–79. 10.1016/S1875-9572(10)60014-9 . 20417456

[27] Taylor SA, Hergenroeder AC. Waist circumference predicts increased cardiometabolic risk in normal weight adolescent males. Int J Pediatr Obes. 2011;6(2–2):e307–11. Epub 2011/06/09. 10.3109/17477166.2011.575149 . 21649469

[28] Lee S, Bacha F, Arslanian SA. Waist circumference, blood pressure, and lipid components of the metabolic syndrome. J Pediatr. 2006;149(6):809–16. Epub 2006/12/02. 10.1016/j.jpeds.2006.08.075 . 17137898

[29] Savva SC, Tornaritis M, Savva ME, Kourides Y, Panagi A, Silikiotou N, et al Waist circumference and waist-to-height ratio are better predictors of cardiovascular disease risk factors in children than body mass index. Int J Obes Relat Metab Disord. 2000;24(11):1453–8. Epub 2000/01/11. . 11126342 10.1038/sj.ijo.0801401

[30] Freedman DS, Serdula MK, Srinivasan SR, Berenson GS. Relation of circumferences and skinfold thicknesses to lipid and insulin concentrations in children and adolescents: the Bogalusa Heart Study. Am J Clin Nutr. 1999;69(2):308–17. Epub 1999/02/16. . 9989697 10.1093/ajcn/69.2.308

[31] Bauer KW, Marcus MD, El Ghormli L, Ogden CL, Foster GD. Cardio-metabolic risk screening among adolescents: understanding the utility of body mass index, waist circumference and waist to height ratio. Pediatr Obes. 2015;10(5):329–37. 10.1111/ijpo.267 25515620 PMC4470887

[32] Alberti KG, Zimmet P, Shaw J. The metabolic syndrome—a new worldwide definition. Lancet. 2005;366(9491):1059–62. Epub 2005/09/27. 10.1016/s0140-6736(05)67402-8 . 16182882

[33] Weiss R, Dziura J, Burgert TS, Tamborlane WV, Taksali SE, Yeckel CW, et al Obesity and the metabolic syndrome in children and adolescents. N Engl J Med. 2004;350(23):2362–74. Epub 2004/06/04. 10.1056/NEJMoa031049 . 15175438

[34] de Ferranti SD, Gauvreau K, Ludwig DS, Neufeld EJ, Newburger JW, Rifai N. Prevalence of the metabolic syndrome in American adolescents: findings from the Third National Health and Nutrition Examination Survey. Circulation. 2004;110(16):2494–7. Epub 2004/10/13. 10.1161/01.cir.0000145117.40114.c7 . 15477412

[35] Ng VW, Kong AP, Choi KC, Ozaki R, Wong GW, So WY, et al BMI and waist circumference in predicting cardiovascular risk factor clustering in Chinese adolescents. Obesity (Silver Spring). 2007;15(2):494–503. Epub 2007/02/15. 10.1038/oby.2007.588 . 17299123

[36] Poirier P, Giles TD, Bray GA, Hong Y, Stern JS, Pi-Sunyer FX, et al Obesity and cardiovascular disease: pathophysiology, evaluation, and effect of weight loss: an update of the 1997 American Heart Association Scientific Statement on Obesity and Heart Disease from the Obesity Committee of the Council on Nutrition, Physical Activity, and Metabolism. Circulation. 2006;113(6):898–918. 10.1161/CIRCULATIONAHA.106.171016 . 16380542

[37] Schroder H, Ribas L, Koebnick C, Funtikova A, Gomez SF, Fito M, et al Prevalence of abdominal obesity in Spanish children and adolescents. Do we need waist circumference measurements in pediatric practice? PLoS One. 2014;9(1):e87549 10.1371/journal.pone.0087549 24475305 PMC3903726

